# Evaluation of the performance of new sticky pots for outdoor resting malaria vector surveillance in western Kenya

**DOI:** 10.1186/s13071-019-3535-3

**Published:** 2019-05-31

**Authors:** Teshome Degefa, Delenasaw Yewhalaw, Guofa Zhou, Ming-Chieh Lee, Harrysone Atieli, Andrew K. Githeko, Guiyun Yan

**Affiliations:** 1 School of Medical Laboratory Sciences, Faculty of Health Sciences, Jimma University, Jimma, Ethiopia; 2 Centre for Global Health Research, Kenya Medical Research Institute, Kisumu, Kenya; 3 Tropical and Infectious Diseases Research Center (TIDRC), Jimma University, Jimma, Ethiopia; 4 Program in Public Health, College of Health Sciences, University of California at Irvine, Irvine, CA 92697 USA; 5 School of Public Health and Community Development, Maseno University, Kisumu, Kenya

**Keywords:** Malaria vectors, Outdoor resting, Vector surveillance, Sticky pot, Kenya

## Abstract

**Background:**

Surveillance of outdoor resting malaria vector populations is crucial to monitor possible changes in vector resting and feeding behaviour following the widespread use of indoor-based vector control interventions. However, it is seldom included in the routine vector surveillance system in Africa due to lack of well standardized and efficient traps. This study was conducted to evaluate the performance of sticky pots for outdoor resting malaria vector surveillance in western Kenya.

**Methods:**

Mosquito collections were conducted from September 2015 to April 2016 in Ahero and Iguhu sites, western Kenya using sticky pots, pit shelters, clay pots, exit traps, Prokopack aspirator and CDC light traps (outdoor and indoor). Species within *Anopheles gambiae* (*s.l.*) were identified using polymerase chain reaction (PCR). Enzyme-linked immunosorbent assay (ELISA) was used to determine blood meal sources of malaria vectors.

**Results:**

A total of 23,772 mosquitoes were collected, of which 13,054 were female anophelines comprising *An. gambiae* (*s.l.*) (72.9%), *An. funestus* (13.2%), *An. coustani* (8.0%) and *An. pharoensis* (5.9%). Based on PCR assay (*n* = 672), 98.6% *An. arabiensis* and 1.4% *An. gambiae* (*s.s.*) constituted *An. gambiae* (*s.l.*) in Ahero, while this was 87.2% *An. gambiae* (*s.s.*) and 12.8% *An. arabiensis* in Iguhu. The sticky pots and pit shelters showed similar performance with regard to the relative abundance and host blood meal indices of *An. gambiae* (*s.l.*) and *An. funestus*. In terms of density per trap, a pit shelter caught on average 4.02 (95% CI: 3.06–5.27) times as many *An. gambiae* (*s.l.*) as a sticky pot, while a sticky pot captured 1.60 (95% CI: 1.19–2.12) times as many *An. gambiae* (*s.l.*) as a clay pot. Exit traps yielded a significantly lower number of *An. gambiae* (*s.l.*) than all other traps in Ahero, but a higher number of *An. gambiae* (*s.l.*) compared to the other outdoor traps in Iguhu. Indoor CDC light traps captured a significantly higher number of *An*. *funestus* than other traps.

**Conclusions:**

Sticky pots could be a useful and complementary tool for outdoor resting malaria vector surveillance, in settings where using pit shelters is not feasible and less productive. The lower vector density in the sticky pots compared to pit shelters suggests that batches of sticky pots (i.e. four per compound) need to be deployed in order to make a direct comparison. This study also highlighted the need to concurrently undertake indoor and outdoor vector surveillance to better understand residual malaria transmission.

## Background

Surveillance of adult malaria vectors is a prerequisite to determine vector density, species composition, behaviour and sporozoite infection rates for surveillance driven control and to evaluate the impact of control interventions. The surveillance tools and procedures usually differ depending on the type of entomological indices to be measured, such as vector biting behaviour, blood meal sources, resting habits or malaria transmission intensity [1]. The vector species may occur as indoor host-seeking, indoor resting, outdoor host-seeking and outdoor resting fractions, each requiring different surveillance tools and approaches [2].

In most African countries, malaria vector surveillance activities rely mainly on sampling host-seeking and indoor resting mosquitoes. The most commonly used methods for sampling host-seeking vectors are human landing catches (HLC) and Centers for Disease Control and Prevention (CDC) light traps [3]. Indoor resting vectors are often sampled by pyrethrum spray catches (PSCs) and indoor aspiration using a Prokopack aspirator [4] or backpack aspirator [5]. Yet, outdoor resting vector sampling is seldom included in the routine vector surveillance system due to lack of well standardized and efficient traps.

However, data from outdoor resting collections is also crucial to monitor possible changes in vector resting and feeding behaviour following the widespread use of indoor-based vector control interventions [6]. This is particularly important in Africa where there is an increasing shift in vector species composition from anthropophagic, endophilic vectors to zoophagic, exophilic sibling species following the wide scale use of insecticide-treated nets (ITNs) and indoor residual spraying (IRS) [7–11]. Such shifts in vector resting behaviour may also occur within vector species, as evidenced by an increased exophilic tendency in *An. gambiae* (*s.s.*) under the influence of insecticide use in houses in western Kenya [12]. Such behavioral shifts could pose a problem on control efforts as the current interventions (ITNs and IRS) do not target outdoor and early indoor biting vectors which eventually rest outdoors to escape from contact with insecticide-treated surfaces and sustain residual malaria transmission [13].

Traditionally, mechanical aspiration of mosquitoes from their natural resting sites such as vegetation, cracks on stone walls, holes in rocks and crevices in the ground or artificial pit-shelters has been used as a method for sampling outdoor resting malaria vectors [14, 15]. Pit shelters have the advantage of providing concentrated sites for collections and representative samples that can be used for quantitative work [6]. However, sampling inside pits is difficult to standardize. It is also difficult to maintain pit shelters, especially during the rainy season as the pits could be saturated with water. Moreover, dangerous animals such as snakes may also be encountered in the pits, causing a risk to mosquito collectors. Last but not least, pits cannot be moved and cannot be deployed in large numbers, which limits its deployment as a general routine surveillance tool.

Recently, alternative sampling tools such as clay pots and resting boxes have also been developed for similar purpose [16–18]. The advantage of these tools is that they are small and portable so that they could be deployed in large numbers and in different settings. Although clay pots have been shown to have good performance when used in batches (i.e. six pots per compound) [16], retrieving mosquitoes resting within the pots needs active aspiration by collectors which may lead to collection bias due to variation in skill among collectors. Moreover, mosquitoes could escape at any time before collection when the pots are disturbed by animals or children playing in the area. Hence, there is a need to develop and standardize tool for outdoor resting malaria vector surveillance.

The aim of this study was thus to evaluate new sticky pots for outdoor resting malaria vector surveillance. The trapping efficiency of the sticky pots was compared with pit shelters, clay pots, window exit traps and Prokopack aspirator in western Kenya. Moreover, CDC light traps were employed in this study to assess whether mosquito species composition and diversity in the outdoor resting collections (by sticky pots, pit shelters and clay pots) are similar with that of host-seeking vector collections.

## Methods

### Study sites

**Fig. 1:**
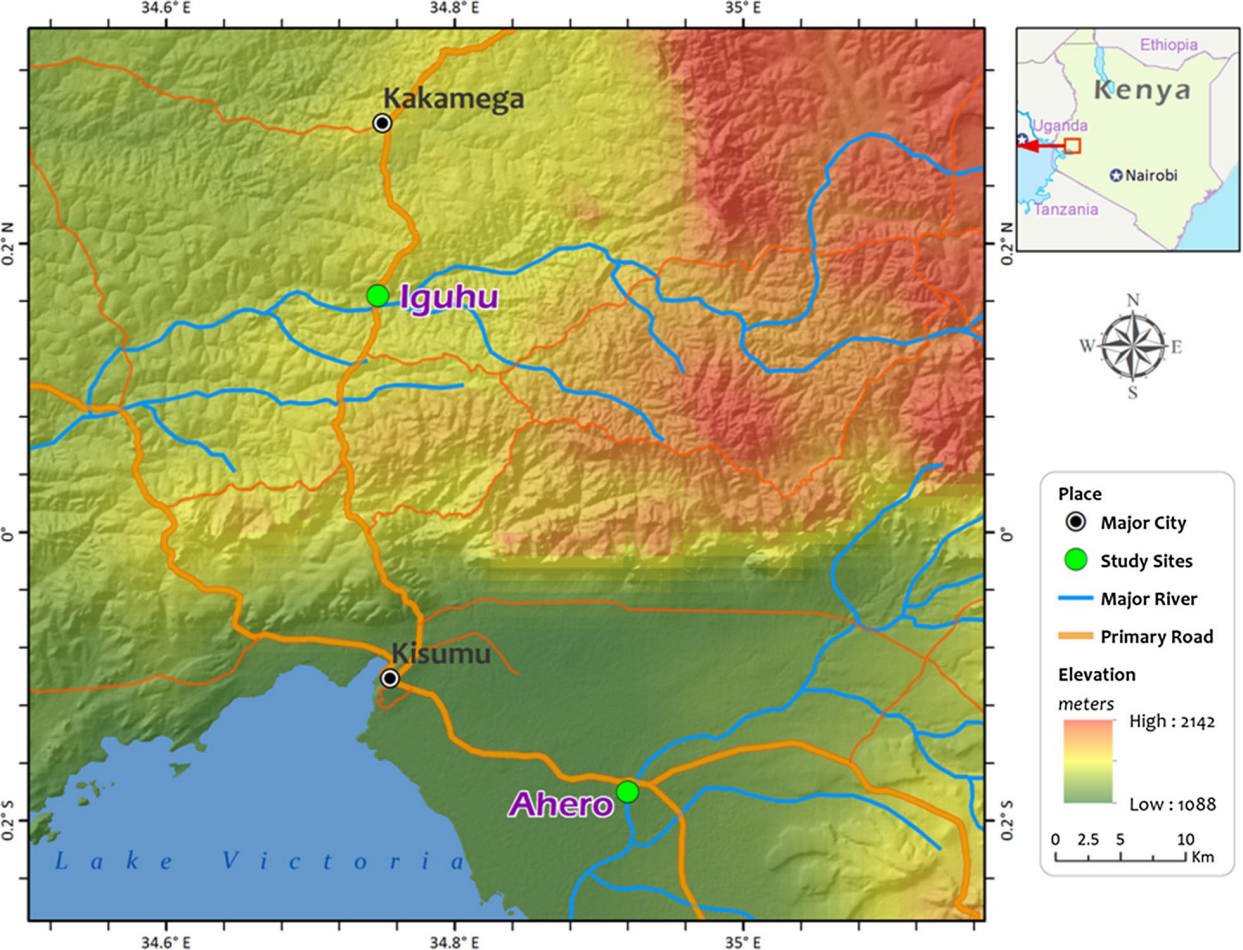
Map of the study sites

The study was conducted in Ahero (00°07′54″S, 34°56′24″E, altitude 1162 meters above sea level, masl) and Iguhu (00°09′35″N; 34°44′46″E, altitude 1430–1580 masl) sites in western Kenya (Fig. 1). Ahero is a lowland plain area located in Kisumu County, while Iguhu is highland with flat-bottomed valleys in Kakamega County. The sites have a bimodal pattern of rainfall, with the long rainy season from April to June, which triggers the peak malaria transmission period and the short rainy season from October to November with minimal transmission [19]. The hot and dry season is from January to March [20]. *Plasmodium falciparum* is the predominant malaria species in the area and is transmitted by *Anopheles gambiae* (*sensu stricto*), *An. arabiensis* and *An. funestus* group [20–22].

### Description of trapping methods

#### Pit shelters

**Fig. 2:**
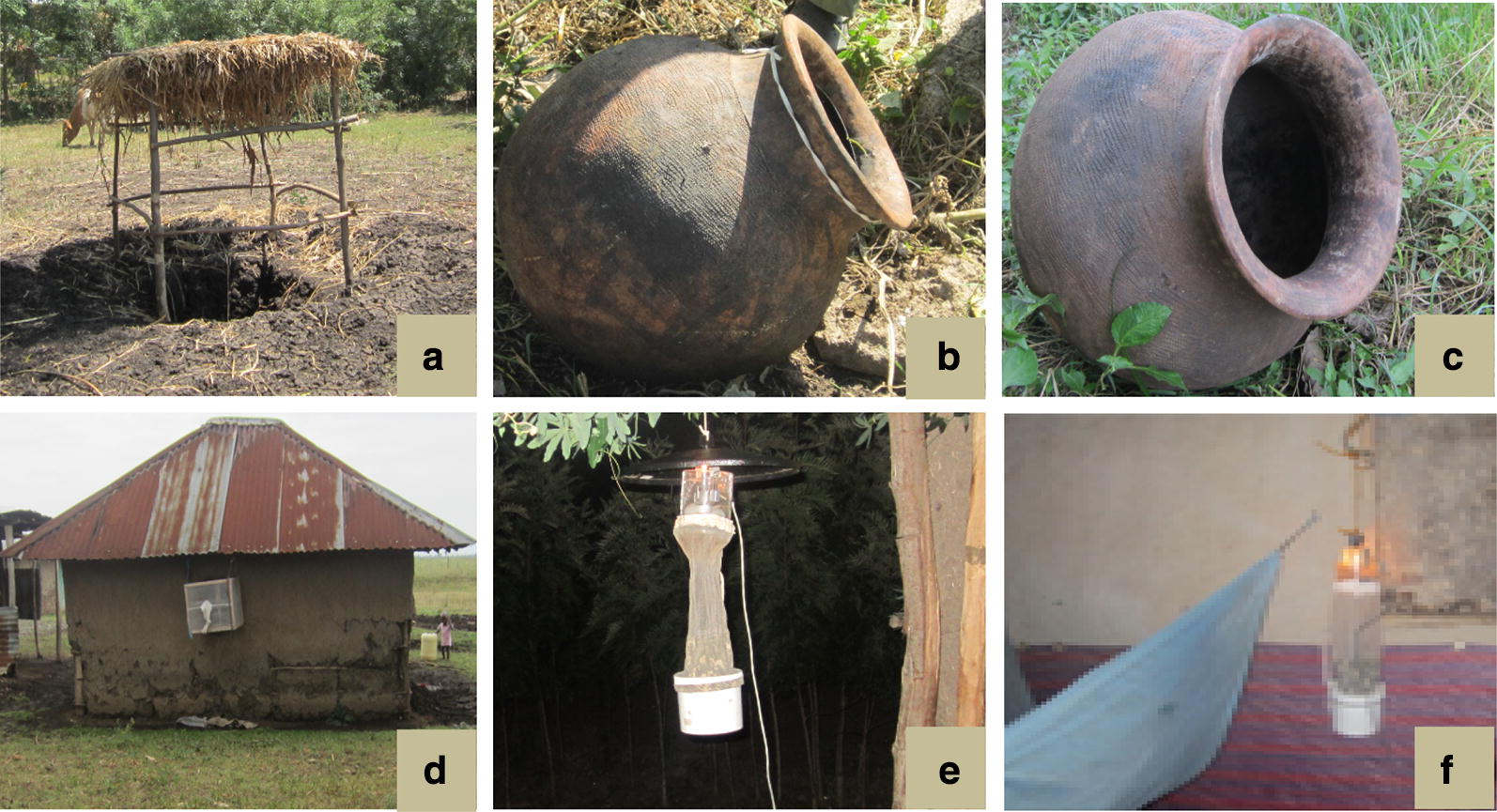
Vector sampling tools [pit shelter (**a**), sticky pot (**b**), clay pot (**c**), exit trap (**d**), outdoor CDC light trap (**e**) and indoor CDC light trap (**f**)] used for outdoor and/or indoor resting/host-seeking malaria vector surveillance in Ahero and Iguhu sites, western Kenya (pictures captured in the field)

A rectangular pit was dug in the ground (1.5 m in depth, 1.2 m in length and 1 m in width) within 20 m of each selected house (Fig. 2a). In each of the four vertical sides, about 50–60 cm and 90–100 cm from the bottom of the pit, two little cavities were dug in to a depth of about 30 cm. The main pits were then shaded by an artificial framework thatched with locally available reeds. Resting mosquitoes were sampled from 06:00 to 09:00 h inside the eight cavities by using hand-held mouth aspirators and an intensive visual search.

#### Sticky pots

Sticky pots are sticky variants of clay pots that have been used previously to collect outdoor resting *Anopheles* mosquitoes [16]. Each sticky pot has an opening of 20 cm width, a round bottom, and a maximum width of 45 cm. The internal surface of the pots was covered with waterproof black papers coated with Tangle-Trap sticky substance (Fig. 2b). This modification was done based on the assumption that covering the internal wall of clay pots with waterproof sticky paper would trap every mosquito that rests within the pot, not only the fractions present at the time of collection. The sticky pots were placed outdoors from 18:00 to 06:00 h to trap resting mosquitoes. Trapped mosquitoes were collected from the sticky pots using forceps from 06:00 to 09:00 h in the morning following each sampling night.

#### Clay pots

Pots similar to sticky pots but without the sticky substance were used (Fig. 2c). The pots were placed outdoors from 18:00 to 06:00 h. Mosquitoes were collected from the pots once in the morning from 06:00 to 09:00 h as follows. White mesh from a mosquito cage was carefully placed over the mouth of the pot and secured as described by Odiere et al. [16]. The collector then lifted the pot and agitated mosquitoes inside the pot, causing them to fly and move into the cage. The mesh was then removed, and any remaining mosquitoes in the pot were retrieved using an aspirator and transferred to a labeled paper cup. Mosquitoes were finally collected from the cage using aspirator and transferred to the paper cup, completing the collection.

#### Window exit trap

Exit traps are rectangular boxes made of a wooden frame covered with netting material, with a slit-shaped rectangular tilted wire opening at one side as a mosquito entrance and a sealable cotton sleeve aspirator inlet on the other side. The trap was set on a window of each of the selected houses every evening at 18:00 h (Fig. 2d). Mosquitoes were retrieved from the trap using a hand-held aspirator through a sealable sleeve in the morning from 06:00 to 09:00 h.

#### Prokopack aspirator

The Prokopack aspirator (John W. Hock, Gainesville, FL, USA) is a recently developed tool for sampling indoor resting mosquitoes [4]. The aspirator is powered by a 12V battery. Indoor resting mosquito collection using a Prokopack aspirator from selected houses was performed every morning concurrently with that of outdoor sampling. Mosquitoes resting on the walls and the area under the roof of the houses or ceilings were systematically aspirated by using progressive downward and upward movements along the wall surfaces of the room.

#### CDC miniature light traps

CDC miniature light traps (John W. Hock) were set inside selected houses near an occupied bed at a height of 1.5 m from 18:00 to 06:00 h in the night to collect indoor host seeking mosquitoes. For the outdoor host-seeking mosquito sampling, a CDC light trap was also set in the vicinity (within 2 m) of sentinel houses from 18:00 to 06:00 h.

### Experimental design

Each study site was classified into ten clusters. A cluster was defined as group of houses closely located on a similar topography. Two houses, approximately 50 m apart, were randomly selected from each cluster, hence a total of 20 houses were selected per site. In each cluster, the two houses were numbered as H1 and H2. One of the two houses was then used for the following combination of trapping methods: one sticky pot and one clay pot placed outdoor at about 5 m from the house, an exit trap set on window, sampling from a pit shelter located within 20 m from the house and indoor aspiration was carried out using Prokopack aspirator. The second house was used for setting CDC light traps (one indoors and one outdoors). In each cluster, the trapping methods were swapped between the two houses for two consecutive days every month. Mosquito collections were conducted during the short rainy season (September to November) in 2015 and dry season (February to April) in 2016. A total of 120 trap-nights were done for each trapping method in each study site.

### Sample processing

All collected mosquitoes were identified morphologically to species or species complexes using keys [23]. Female *Anopheles* mosquitoes were further classified as unfed, freshly fed, half-gravid and gravid. Each female *Anopheles* mosquito was then kept in a labeled 1.5 ml Eppendorf tube with cotton wool over silica gel desiccant. Samples were stored in a − 20 °C freezer at the Climate and Human Health Research Laboratory of Kenya Medical Research Institute (KEMRI) until used for further processing.

### Molecular identification of vector species complexes

Members of *An. gambiae* (*sensu lato*) and *An. funestus* group were identified to species by polymerase chain reaction (PCR), following the protocols developed by Scott et al. [24] for *An. gambiae* (*s.l.*) and Koekemoer et al. [25] for *An. funestus* group.

### Detection of blood meal sources

The blood meal sources of blood fed *Anopheles* mosquitoes were analyzed by a direct enzyme-linked immunosorbent assay (ELISA) using human, bovine, goat, chicken and dog antibodies [26]. Positive controls were included for each host during the assay. Laboratory reared unfed *An. gambiae* was used as negative control.

### Data analysis

The relative abundance of anopheline mosquitoes collected by each trap was determined as the percent composition of each anopheline species relative to the total number of anophelines captured. A Chi-square test was used to compare the difference in *Anopheles* mosquito species composition among the trapping methods. The difference in *Anopheles* mosquito density among different trapping methods was compared using a generalized linear model (GLM) based on a negative binomial distribution. Sampling season was treated as a covariate in the model. The estimated marginal mean (EMM) density of *Anopheles* mosquitoes was determined for each trap using negative binomial regression by adjusting for season. Pairwise comparison of different traps in terms of the EMM of *Anopheles* mosquitoes was also performed using negative binomial regression model.

Gini-Simpson’s diversity index (1-D) [27–29] was applied to evaluate mosquito species diversity for each trap. To determine the statistical significance of difference in species diversity among the traps, 95% confidence intervals (CI) were calculated [30]. Simpson’s index of evenness (E) was calculated to obtain a measure of the relative abundance of the different species in the sample [27, 31].

The human blood index (HBI) was calculated as the number of *Anopheles* mosquitoes that fed on human over the total number of *Anopheles* tested for blood meal origins multiplied by a hundred [32]. The bovine blood index (BBI) and blood meal indices of other hosts (goat, dog and chicken) were also determined in a similar way. Mixed blood meals were included in the calculation of blood meal indices [33]. A Chi-square test was used to compare host blood meal indices of malaria vectors between different trapping methods.

Data were analyzed using R v.3.3 (R Core Team) and SPSS v.20.0 (SPSS, Chicago, IL, USA) software packages. *P* < 0.05 was considered statistically significant during the analysis.

## Results

### Species composition and abundance

A total of 23,772 mosquitoes were collected during the study period (Table 1): 5847 (24.6%) from pit shelters, 1627 (6.8%) by sticky pots, 1249 (5.3%) by clay pots, 6311 (26.6%) by outdoor CDC light traps, 1400 (5.9%) by exit traps, 2715 (11.4%) from indoors by Prokopack aspirator and 4623 (19.4%) by indoor CDC light traps. The majority (74.9%) of the collected mosquitoes were anophelines, while the remaining 25.1% were *Culex* species. Most (89.3%) of the mosquitoes were collected from the Ahero site. Of the 17,807 anopheline mosquitoes collected, 73.3% (*n* = 13,054) were female anophelines. *Anopheles gambiae* (*s.l.*) was the predominant species accounting for 72.9% of the total female *Anopheles* mosquitoes collected, followed by *An. funestus* group (13.2%), *An. coustani* (8.0%) and *An. pharoensis* (5.9%).

**Table 1:** Summary of mosquitoes collected by different trapping methods in Ahero and Iguhu sites, western Kenya (*n* = 120 trap-nights per site for each trap)

Site and species	Sex	Outdoors					Indoors		Total
		Pit shelter	Sticky pot	Clay pot	Light trap	Exit trap	Prokopack	Light trap	
Ahero									
*An. gambiae* (*s.l.*)	Female	3262	706	510	1636	336	1031	1592	9073
	Male	1876	634	501	210	168	551	178	4118
*An. funestus* group	Female	142	28	16	270	380	135	628	1599
	Male	72	24	18	26	35	108	7	290
*An. coustani*	Female	15	2	0	652	41	3	321	1034
	Male	1	0	0	8	1	0	4	14
*An. pharoensis*	Female	0	0	0	688	1	0	78	767
	Male	0	1	0	42	0	0	2	45
*Culex* spp.	Female	88	51	30	2044	90	59	1064	3426
	Male	79	32	38	463	16	27	214	869
Iguhu									
*An. gambiae* (*s.l.*)	Female	41	9	7	56	159	57	108	437
	Male	86	37	34	4	29	37	7	234
*An. funestus* group	Female	4	3	2	13	17	42	49	130
	Male	19	1	1	0	11	15	3	50
*An. coustani*	Female	0	0	0	10	0	1	3	14
	Male	1	1	0	0	0	0	0	2
*Culex* spp.	Female	101	53	44	70	53	399	142	862
	Male	60	45	48	119	63	250	223	808
Total		5847	1627	1249	6311	1400	2715	4623	23,772

**Fig. 3:**
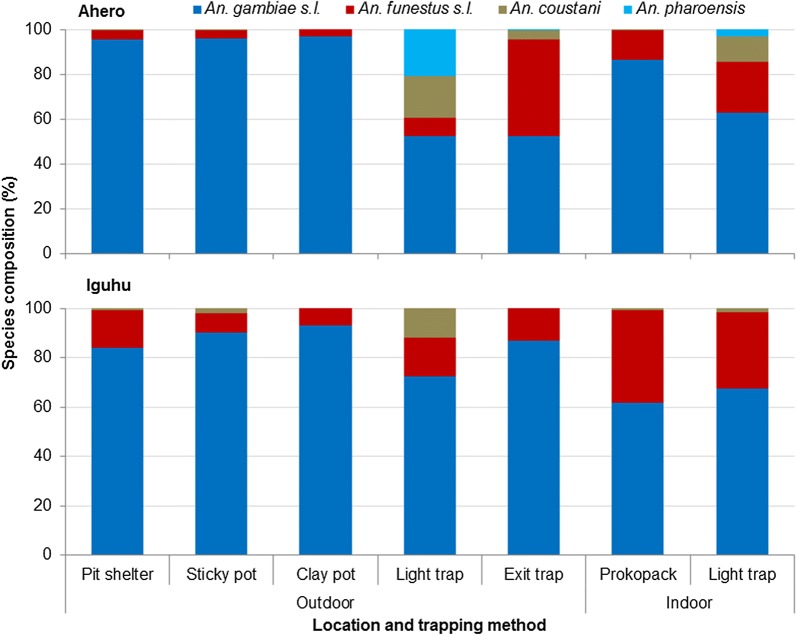
The relative abundance of female *Anopheles* mosquitoes collected by different trapping methods in Ahero and Iguhu sites, western Kenya

Figure 3 shows the relative abundance of *Anopheles* mosquitoes collected by different trapping methods. The relative abundance of *Anopheles* species collected by the sticky pots was similar with that of pit shelters (*χ*^2^ = 0.429, *df* = 2, *P* = 0.807) and clay pots (*χ*^2^ = 3.21, *df* = 2, *P* = 0.201), *An. gambiae* (*s.l.*) being the most predominant species accounting for 95.9, 95.4 and 96.6% of the anophelines collected by the sticky pots, pit shelters and clay pots, respectively. However, there was significant difference between outdoor and indoor traps, i.e. pit shelters *versus* Prokopack aspirator (*χ*^2^ = 139, *df* = 2, *P* < 0.001) and outdoor CDC light traps *versus* indoor CDC light traps (*χ*^2^ = 720, *df* = 3, *P* < 0.001). For instance, the proportion of *An. funestus* group was 15.2% by Prokopack aspirator, while it was 3.9, 4.3 and 3.4% by sticky pots, pit shelters and clay pots, respectively. Similarly, *An. funestus* group accounted for 23.1% of the anopheline species collected by indoor CDC light traps, while it was 8.5% by outdoor CDC light traps.

### Species diversity

Mosquito species diversity was significantly higher from sticky pots (Simpson diversity index ± SD, 0.26 ± 0.03) than pit shelters (0.18 ± 0.02), but in both traps mosquito species diversity was lower as compared to outdoor CDC light traps (0.70 ± 0.01), exit traps (0.63 ± 0.01), Prokopack aspirator (0.53 ± 0.02) and indoor CDC light traps (0.68 ± 0.01) (Table 2). There was no significant difference in mosquito species diversity between collections from sticky pots and clay pots. Outdoor CDC light traps collected mosquitoes of different species more evenly (Simpson’s evenness index of 0.87) than the other traps, while the species evenness of mosquitoes collected in pit shelters (evenness index of 0.25) and sticky pots (evenness index of 0.32) were relatively lower compared to other traps.

**Table 2:** Comparison of mosquito species diversity among different trapping methods, western Kenya

Place of collection	Trapping method	Species richness	Simpson’s diversity index, 1-D (95% CI)	Simpson’s evenness, E
Outdoors	Pit shelter	4	0.18 (0.17–0.20)^a^	0.25
	Sticky pot	5	0.26 (0.23–0.29)^b^	0.32
	Clay pot	3	0.27 (0.24–0.30)^b^	0.37
	Light trap	5	0.70 (0.69–0.71)^d^	0.87
	Exit trap	5	0.63 (0.62–0.64)^c^	0.79
Indoors	Prokopack	4	0.53 (0.52–0.55)^e^	0.71
	Light trap	5	0.68 (0.67–0.69)^f^	0.85

*Note*: The different superscript letters indicate that mosquito species diversity varied significantly between trapping methods

### Mosquito density

The density of female *Anopheles* mosquitoes varied among different traps (Tables 3, 4). In Ahero, pit shelters yielded a significantly higher number of *An. gambiae* (*s.l.*) (EMM density per pit = 24.26, 95% CI: 19.79–28.73) than all other traps (*P* < 0.05). After adjusting for season, a pit shelter caught on average 4.02 (95% CI: 3.06–5.27) and 6.37 (95% CI: 4.83–8.41) times as many *An. gambiae* (*s.l*.) per day as a sticky pot and clay pot, respectively. Similarly, pit shelters were 2.95 (95% CI: 2.26–3.87), 10.21 (95% CI: 7.67–13.60), 3.19 (95% CI: 2.44–4.16) and 2.96 (95% CI: 2.26–3.87) times more likely to collect *An. gambiae* (*s.l.*) compared to outdoor CDC light traps, exit traps, Prokopack aspirator and indoor CDC light traps, respectively. The mean density of *An. gambiae* (*s.l.*) was significantly higher in sticky pots than clay pots and exit traps (*P* < 0.05). A sticky pot caught 1.60 (95% CI: 1.19–2.12) and 2.54 (95% CI: 1.89–3.42) times as many *An. gambiae* (*s.l*.) as a clay pot and an exit trap, respectively. The difference in mean *An. gambiae* (*s.l.*) between indoor and outdoor CDC light traps was not significant (*P* = 0.986).

**Table 3:** Estimated marginal mean density for female *An. gambiae* (*s.l.*) and *An. funestus* group in Ahero and Iguhu sites, western Kenya

Site and species	Outdoors					Indoors	
	Pit shelter	Sticky pot	Clay pot	Light trap	Exit trap	Prokopack	Light trap
Ahero							
*An. gambiae* (*s.l.*)	24.26 (19.79–28.73)^a^	6.03 (4.82–7.25)^b^	3.81 (3.02–4.59)^c^	8.21 (6.63–9.80)^c^	2.38 (1.85–2.89)^d^	7.62 (6.14–9.09)^b,c^	8.19 (6.61–9.77)^c^
*An. funestus* group	0.79 (0.58–1.00)^a^	0.16 (0.09–0.23)^b^	0.09 (0.04–0.14)^b^	1.77 (1.36–2.19)^c^	1.86 (1.44–2.28)^c^	0.74 (0.54–0.94)^a^	4.59 (3.64–5.54)^d^
Iguhu							
*An. gambiae* (*s.l*.)	0.33 (0.21–0.45)^a^	0.07 (0.02–0.12)^b^	0.05 (0.01–0.10)^b^	0.46 (0.31–0.61)^a^	1.20 (0.91–1.49)^c^	0.45 (0.31–0.59)^a^	0.91 (0.67–1.15)^c^
*An. funestus* group	0.03 (0.001–0.06)^a^	0.02 (0.00–0.05)^a^	0.02 (0.00–0.04)^a^	0.11 (0.04–0.17)^b^	0.14 (0.07–0.21)^b^	0.33 (0.21–0.45)^c^	0.40 (0.26–0.53)^c^

*Note*: For each study site, across each row, the different letters indicate that the estimated marginal mean density varied significantly (*P* < 0.05). The estimated marginal means were determined using negative binomial regression model by adjusting for season

**Table 4:** Estimates of a negative binomial regression for comparison of vector density between pit shelter and other trapping methods in western Kenya

Species and place of collection	Trapping method	Ahero		Iguhu	
		Exponentiated estimate (OR)	*P*-value	Exponentiated estimate (OR)	*P*-value
*An. gambiae* (*s.l.*)					
Outdoors	Pit shelter	1.0^a^		1.0^a^	
	Sticky pot	0.25 (0.20–0.33)	< 0.001	0.22 (0.10–0.47)	< 0.001
	Clay pot	0.16 (0.12–0.20)	< 0.001	0.17 (0.07–0.39)	< 0.001
	Light trap	0.34 (0.26–0.44)	< 0.001	1.40 (0.86–2.27)	0.173
	Exit trap	0.10 (0.07–0.13)	< 0.001	3.65 (2.37–5.61)	< 0.001
Indoors	Prokopack	0.31 (0.24–0.41)	< 0.001	1.37 (0.85–2.21)	0.199
	Light trap	0.34 (0.26–0.44)	< 0.001	2.76 (1.77–4.30)	< 0.001
*An. funestus* group					
Outdoors	Pit shelter	1.0^a^		1.0^a^	
	Sticky pot	0.20 (0.122–0.33)	< 0.001	0.75 (0.17–3.35)	0.716
	Clay pot	0.12 (0.07–0.21)	< 0.001	0.50 (0.09–2.80)	0.433
	Light trap	2.25 (1.58–3.21)	< 0.001	3.27 (1.04–10.33)	0.044
	Exit trap	2.36 (1.68–3.32)	< 0.001	4.37 (1.43–13.40)	0.010
Indoors	Prokopack	0.94 (0.64–1.36)	0.726	10.37 (3.60–29.88)	< 0.001
	Light trap	5.83 (4.14–8.20)	< 0.001	12.33 (4.3–35.30)	< 0.001

^a^Reference value

*Abbreviation*: OR-odds ratio

In Iguhu on the other hand, the mean density of *An. gambiae* (*s.l.*) was significantly higher from exit traps than all other traps except indoor CDC light traps. The mean density of *An. gambiae* (*s.l.*) was significantly higher from pit shelters as compared to sticky pots and clay pots, whereas the difference in mean density of *An. gambiae* (*s.l.*) between pit shelters and Prokopack aspirator was not significant (*P* = 0.20). The mean density of *An. gambiae* (*s.l.*) was significantly higher from indoor CDC light traps than outdoor CDC light traps (Table 3).

The mean density of *An. funestus* group was significantly higher from indoor CDC light traps than the other traps in both sites. In Ahero, pit shelters captured higher density of *An. funestus* group than sticky pots and clay pots, whereas in Iguhu the mean density of *An. funestus* group did not vary significantly among the three traps (*P* > 0.05) (Table 3).

### Composition of *An. gambiae* and *An. funestus* species complexes

**Fig. 4:**
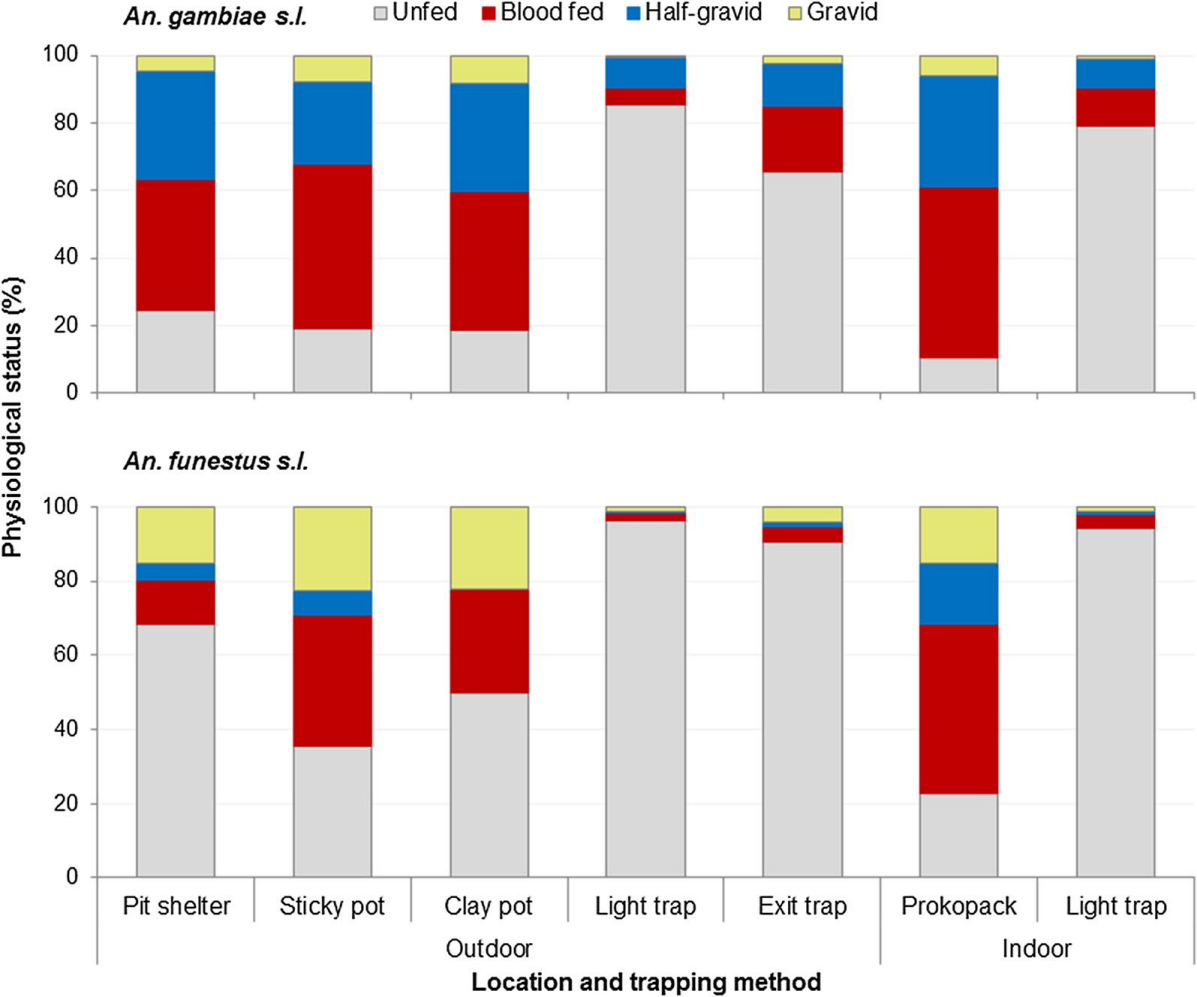
Physiological status of *An. gambiae* (*s.l.*) and *An. funestus* group collected by different trapping methods, western Kenya

A total of 872 specimens [738 *An. gambiae* (*s.l.*) and 134 *An. funestus* group] from different traps were analysed for identification of sibling species. Of these, 672 *An. gambiae* (*s.l.*) and 110 *An. funestus* group specimens were successfully amplified and identified to species using species specific PCR. Figure 4 shows member species of *An. gambiae* (*s.l.*). In Ahero, of the *An. gambiae* (*s.l.*) specimens assayed, *An. arabiensis* and *An. gambiae* (*s.s.*) accounted for 98.6 and 1.4%, respectively. The proportion of *An. arabiensis* was 100.0% from pit shelters, sticky pots, clay pots and outdoor CDC light traps, while it was 92.9, 96.5 and 97.4% in exit traps, Prokopack aspirator and indoor CDC light traps, respectively. In Iguhu, of the *An. gambiae* (*s.l.*) specimens assayed, *An. arabiensis* and *An. gambiae* (*s.s.*) accounted for 12.8 and 87.2%, respectively. Overall, *An. gambiae* sibling species composition did not vary significantly between pit shelters and sticky pots (*χ*^2^ = 0.018, *df* = 1, *P* = 0.894), pit shelters and clay pots (*χ*^2^ = 0.122, *df* = 1, *P* = 0.727); however, there was a significant difference in species composition between collections from pit shelters and other traps (*P* < 0.001). Of the amplified *An. funestus* group specimens, *Anopheles funestus* (*s.s.*) (hereafter *An. funestus*) and *An. leesoni* accounted for 98.2 and 1.8%, respectively. The sibling species composition of *An. funestus* group did not vary significantly among different traps (*χ*^2^ = 5.69, *df* = 6, *P* = 0.459).

### Physiological status

**Fig. 5:**
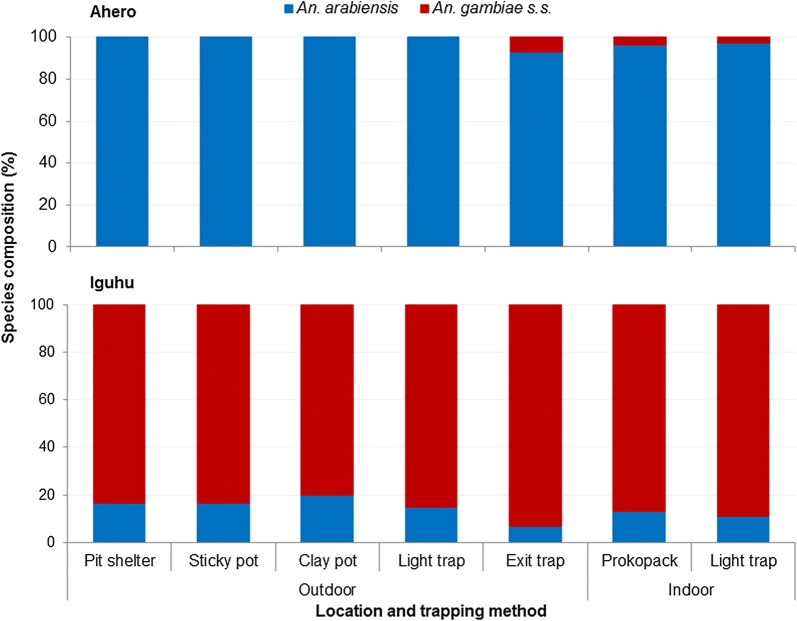
Composition of *An. gambiae* (*s.l.*) sibling species in Ahero and Iguhu sites, western Kenya

Figure 5 shows physiological status of *An. gambiae* (*s.l.*) and *An. funestus.* The physiological status of *An. gambiae* (*s.l.*) varied significantly among different traps (*χ*^2^ = 3510, *df* = 18, *P* = <0.001). Pit shelters, sticky pots, clay pots and Prokopack aspirator yielded a relatively higher proportion of blood-fed *An. gambiae* (*s.l*.), whereas exit traps and CDC light traps captured mostly unfed *An. gambiae* (*s.l.*). Similarly, the physiological status of *An. funestus* varied significantly among the different traps (*χ*^2^ = 694, *df* = 18, *P* < 0.001). Prokopack aspirator yielded higher proportion of blood-fed *An. funestus*, and relatively fewer unfed *An. funestus* than the other traps. Most of the *An. funestus* collected by exit traps (90%) and CDC light traps (> 94%) were unfed.

### Blood meal sources

Table 5 shows the host blood meal indices of malaria vectors collected by different traps. In Ahero, the overall HBI and BBI of *An. arabiensis* was 2.2 and 75.7%, respectively. There was no significant difference between pit shelters and sticky pots in terms of the host blood meal indices of *An. arabiensis* (*χ*^2^ = 0.492, *df* = 2, *P* = 0.782). Similarly, blood meal indices of *An. arabiensis* did not vary significantly between pit shelters, clay pots and exit traps (*P* > 0.05). However, there was significant difference between pit shelters and outdoor CDC light traps (*χ*^2^ = 33.2, *df* = 2, *P* < 0.001), pit shelters and Prokopack aspirator (*χ*^2^ = 14.6, *df* = 2, *P* = 0.001), and pit shelters and indoor CDC light traps (*χ*^2^ = 35.6, *df* = 2, *P* < 0.001) in terms of the blood meal indices of *An. arabiensis*.

**Table 5:** Blood meal indices of malaria vectors collected by different trapping methods in western Kenya

Species	Blood meal index	Outdoors					Indoors		Total
		Pit shelter	Sticky pot	Clay pot	Light trap	Exit trap	Prokopack	Light trap	
*An. arabiensis*	Number tested	298	66	47	59	30	100	122	722
	HBI	0.7	1.5	0	3.4	3.3	1.0	8.2	2.2
	BBI	85.6	84.8	83	50.8	73.3	68.0	62.3	75.7
	GBI	1.3	1.5	2.1	1.7	0	7.0	4.1	2.6
	DBI	3.4	3.1	2.1	1.7	0	2.0	3.3	2.8
	CBI	0.7	0	0	0	0	6.0	1.6	1.4
	Unknown	10.1	10.6	12.8	42.4	23.3	18.0	23.8	17.0
*An. gambiae* (*s.s.*)	Number tested	13	4	3	10	14	16	10	70
	HBI	23.1	25	33.3	20	42.9	75.0	70	45.7
	BBI	46.2	50	66.7	40	14.3	25.0	0	28.6
	GBI	0	0	0	0	0	0	0	0
	DBI	7.7	0	0	0	0	6.3	0	2.9
	CBI	0	0	0	0	0	0	0	0
	Unknown	23.1	25	0	40	42.9	0	30	24.3
*An. funestus*	Number tested	13	10	3	6	7	56	24	119
	HBI	46.2	50	33.3	50	57.1	62.5	62.5	58.0
	BBI	38.5	50	66.7	33.3	14.3	19.6	8.3	23.5
	GBI	0	0	0	0	0	1.8	4.2	1.7
	DBI	7.7	0	0	0	0	1.8	4.2	2.5
	CBI	0	0	0	0	0	0	0	0
	Unknown	7.7	0	0	16.7	28.6	17.9	20.8	16.0

*Note*: HBI was calculated as the proportion (%) of mosquitoes positive for human (including mixed blood meals) out of the total number of mosquitoes tested. Blood meal indices of other hosts were determined in a similar way

*Abbreviations*: HBI, human blood index; BBI, bovine blood index; GBI, goat blood index; DBI, dog blood index; CBI, chicken blood index

In Iguhu, the overall HBI and BBI of *An. gambiae* (*s.s.*) was 45.7 and 28.6%, respectively. There was no significant difference between pit shelters and sticky pots in terms of the host blood meal indices of *An. gambiae* (*s.s.*) (*χ*^2^ = 0.049, *df* = 2, *P* = 0.976). Likewise, the blood meal indices of *An. gambiae* (*s.s.*) did not vary significantly between pit shelters, clay pots, outdoor CDC light traps and exit traps (*P* > 0.05). However, the blood meal indices of *An. gambiae* (*s.s.*) varied significantly between pit shelters and Prokopack aspirator (*χ*^2^ = 7.195, *df* = 2, *P* = 0.027) as well as between pit shelters and indoor CDC light traps (*χ*^2^ = 7.48, *df* = 2, *P* = 0.024). The HBI of *An. gambiae* (*s.s.*) from indoor CDC light traps (70.0%) and Prokopack aspirator (75.0%) was relatively higher than the HBI of *An. gambiae* (*s.s.*) from outdoor traps, i.e. pit shelters (23.1%), sticky pots (25.0%), clay pots (33.3%), outdoor CDC light traps (20.0%) and exit traps (42.9%). On the other hand, the BBI of *An. gambiae* (*s.s.)* from outdoor traps was higher than the BBI of *An. gambiae* (*s.s.*) from indoor traps (Table 5).

The overall HBI and BBI of *An. funestus* was 58.0 and 23.5%, respectively. The host blood meal indices of *An. funestus* did not vary significantly among different traps (*χ*^2^ = 13.24, *df* = 12, *P* = 0.352). Blood meal indices of other hosts (goat, dog and chicken) were low for all anopheline species in all traps.

## Discussion

The results of this study showed that the new sticky pots performed consistently with pit shelters with regard to the relative abundance of anopheline species captured. In both traps, *An. gambiae* (*s.l.*) was the most abundant anopheline species with remarkably similar proportion followed by *An. funestus* group, indicating that the sticky pots could be a useful alternative tool for outdoor resting malaria vector surveillance, substituting pit shelters. Although pit shelters have been considered as a productive tool for sampling outdoor resting vectors [2, 6], digging pits is not practical in many situations, especially during a rainy season since the pits could be filled with water, causing a risk to children and livestock wandering in the area [2].

However, the mean density of anophelines per trap was significantly lower in the stick pots compared to pit shelters. This variation could be due to the difference in the size of the two traps. A pit shelter had eight cavities for mosquito collection with a total volume (~ 12,000 cm^3^/cavity) roughly equivalent to the volume of five sticky pots (~ 20,000 cm^3^/pot). Previous studies have also reported similar findings for traps of smaller size relative to pit shelters. For instance, a pit shelter captured five to eight times as many *An. gambiae* (*s.l.*) as a sticky resting box in Burkina Faso [18]. Similarly, a study done by Odiere et al. [16], in which six clay pots were pooled for each pit shelter, showed that a clay pot actually yielded a lower number of *An. gambiae* (*s.l.*) compared to a pit shelter. In this study, a pit shelter caught on average four times as many *An. gambiae* (*s.l.*) as a sticky pot. This suggests that deploying four sticky pots per compound could replace a pit shelter for sampling outdoor resting *An. gambiae* (*s.l.*). A similar relative catching rate was also recorded for *An. funestus*.

The sticky pots performed better than clay pots in terms of the mean number of outdoor resting *An. gambiae* (*s.l.*) collected per trap. This shows that coating the internal surface of the sticky pots with sticky paper increased their trapping efficiency as compared to clay pots. Actually, the adhesive feature of the sticky pots offers an additional advantage of allowing passive collection of resting mosquitoes compared to clay pots and pit shelters, both of which need active aspiration of resting mosquitoes [2, 16].

Furthermore, the sticky pots have a number of advantages over pit shelters and clay pots. First, sticky pots are a standardized trapping method and not biased by the skill of a collector, while mosquito collection from pits and clay pots relies on the skill of the collector and a fraction of mosquitoes could escape during collection. Secondly, sticky pots are cheaper compared to pit shelters. The cost of making a sticky pot was less than US $4, whereas that of building a pit shelter was more than US $25 for this study. Thirdly, sticky pots are portable and can be rotated to different sites for use unlike pit shelters which are fixed. Moreover, sticky pots are environmentally safe compared to pit shelters which may raise community concern associated with digging the pits in their compound.

The host blood meal indices of anopheline mosquitoes collected by the sticky pots were also similar with that of pit shelters, indicating the importance of the sticky pots for monitoring the feeding behaviour of exophilic anopheline mosquitoes in settings where using pit shelters is not feasible. This could address the problem of outdoor vector surveillance tools in an effort to monitor vector feeding behaviour due to a difficulty of locating adults in highly dispersed outdoor potential resting sites [1, 34]. The sticky pots have the potential to overcome such challenge.

When we compare all the traps deployed in this study, mosquito species diversity and mean density varied significantly between traps of different location (indoors *vs* outdoors). In Ahero, the density of resting *An. arabiensis* was significantly higher in pit shelters than for Prokopack aspirator, whereas in Iguhu, the density of *An. gambiae* (*s.l.*) [87.2% of which were *An. gambiae* (*s.s*.)] was higher from Prokopack aspirator than pit shelters. The density of host-seeking *An. arabiensis* was relatively higher in outdoor than indoor CDC light traps in Ahero, while the mean density of host-seeking *An. gambiae* (*s.s.*) was significantly higher in indoor than outdoor CDC light traps. Such differences could be explained by variations in vector behaviour rather than difference in the catching efficiency between the traps. Populations of *An. arabiensis* are highly exophilic and exophagic, hence more likely to be captured preponderantly outdoors than indoors, whereas *An. gambiae* (*s.s.*) is relatively endophilic and endophagic [12, 35, 36], thus more likely to be efficiently captured indoors than outdoors.

It is worth mentioning that the density of *An. gambiae* (*s.s.*) was significantly higher from exit traps than Prokopack aspirator in both sites. A similar finding was recorded for *An. funestus* in Ahero. This implies that a significant number of these species, most of which were unfed, exited houses. This might verify their endophagic behaviour in normal circumstance, but they could be forced to leave houses before feeding due to high ITN coverage in the study area [22, 37]. While ITN is the main intervention to reduce human vector contact, it could also force previously anthropophagic vectors to adapt feeding on non-human hosts, as has been recently reported for *An. gambiae* (*s.s*.) [37, 38] or shift their biting time as it has been the case for *An. funestus* [39, 40]. Such vector behavioral shifts could hamper malaria control as residual transmission may occur even with high coverage of indoor-based vector control interventions [13]. Hence, vector surveillance is crucial to evaluate the effectiveness of control interventions.

It is important to note that the host blood meal indices of anopheline mosquitoes varied significantly between indoor and outdoor traps even for anophelines of the same species. For instance, the HBI of *An. arabiensis* collected by indoor CDC light traps was two times as high as the HBI of the same species collected by outdoor CDC light traps. The BBI of indoor resting *An. arabiensis* collected by Prokopack aspirator was 68.0%, while the BBI of outdoor resting fractions of *An. arabiensis* collected by pit shelters, sticky pots and clay pots was each about 85%. Similarly, the HBI of indoor resting *An. gambiae* (*s.s.*) was three times as high as the HBI of outdoor resting fraction of *An. gambiae* (*s.s.*), whereas the BBI of outdoor resting *An. gambiae* (*s.s.*) was two times as high as the BBI of indoor resting *An. gambiae* (*s.s.*). Likewise, the HBI of *An. funestus* was relatively higher in indoor collection than outdoor, while its BBI was higher in outdoor collection than indoor. This could be due to the difference in host availability between indoor and outdoor locations which can affect the feeding behaviour of malaria vectors, as reported elsewhere [41, 42]. This highlights the need to sample outdoor resting/host-seeking fractions of vectors concurrently with indoor resting/host-seeking vectors to determine unbiased vector blood meal sources so that changes in vector feeding and resting behaviour can be monitored.

Given that various entomological indices (e.g. vector density, species composition, host preferences, biting and resting behaviour, and infection rate) need to be monitored in a vector surveillance system, no single trapping method can provide a reliable estimate of vector parameters. For a good representation of the resting vector population, indoor resting vector surveillance (using Prokopack aspirator or PSC) needs to be complemented with outdoor resting vector surveillance. The sticky pots are potential tools to be used for routine surveillance of outdoor resting vectors in areas where using pit shelters is not practical. Light traps remain a relevant tool for host-seeking vector surveillance in the absence of HLC.

The limitation of this study is that a single sticky pot was set in each selected compound despite its smaller size as compared to the size of a pit shelter, and comparison was made on one-to-one basis. This may underestimate the number of *Anopheles* mosquitoes collected by the sticky pots.

## Conclusions

The results of this study revealed that sticky pots could be an alternative tool for outdoor resting malaria vector surveillance, in settings where using pit shelters is not feasible. Unlike pit shelters and clay pots which require active aspiration, the sticky pots have an advantage of collecting resting mosquitoes passively without bias. The lower vector density in the sticky pots compared to pit shelters suggests that batches of sticky pots (i.e. four per compound) need to be deployed to make a direct comparison. This study also highlights the need to concurrently undertake outdoor resting/host-seeking and indoor resting/host-seeking vector surveillance to better understand residual malaria transmission.

## Abbreviations

BBI: bovine blood index
CBI: chicken blood index
CDC: Centers for Disease Control and Prevention
CI: confidence interval
DBI: dog blood index
df: degree of freedom
ELISA: enzyme-linked immunosorbent assay
EMM: estimated marginal mean
GBI: goat blood index
HBI: human blood index
HLC: human landing catch
IRS: indoor residual spraying
ITN: insecticide-treated net
PCR: polymerase chain reaction
PSC: pyrethrum spray catch
SD: standard deviation

## Availability of data and materials

Data supporting the conclusions of this article are included within the article. The raw data are available from the corresponding author upon reasonable request.
